# Circulating miR-21, miR-378, and miR-940 increase in response to an acute exhaustive exercise in chronic heart failure patients

**DOI:** 10.18632/oncotarget.6966

**Published:** 2016-01-21

**Authors:** Tianzhao Xu, Qiulian Zhou, Lin Che, Saumya Das, Lemin Wang, Jinfa Jiang, Guanghe Li, Jiahong Xu, Jianhua Yao, Hongbao Wang, Yue Dai, Junjie Xiao

**Affiliations:** ^1^ Regeneration and Ageing Lab, Experimental Center of Life Sciences, School of Life Science, Shanghai University, Shanghai 200444, China; ^2^ Shanghai Key Laboratory of Bio-Energy Crops, School of Life Science, Shanghai University, Shanghai 200444, China; ^3^ Department of Cardiology, Tongji Hospital, Tongji University School of Medicine, Shanghai 200065, China; ^4^ Cardiovascular Division of the Beth Israel Deaconess Medical Center and Harvard Medical School, Boston, MA 02215, USA; ^5^ Department of Cardiology, Yangpu Hospital, Tongji University School of Medicine, Shanghai 200090, China

**Keywords:** microRNA, heart failure, exercise

## Abstract

Congestive heart failure (CHF) is a major cause of hospitalizations, morbidity, and mortality in Western societies. In addition to optimal medical and device therapy, exercise training is an important adjunct treatment option for CHF patients. MicroRNAs (miRNAs, miRs) participate in a variety of physiological and pathological processes. Dynamic regulation of circulating miRNAs during exercise in healthy persons and athletes has recently been documented, however, the response of circulating miRNAs to exercise in CHF patients is undetermined. Twenty-eight CHF patients underwent a symptom-limited incremental cardiopulmonary exercise test on a bicycle ergometer using a standardized exercise protocol of revised Ramp10 programs at Shanghai Tongji Hospital. Blood samples were collected before and immediately after an acute exercise session. RNA was extracted from the serum and selected miRNAs were determined using quantitative polymerase chain reactions. Moreover, inflammatory and muscle damage markers were determined by enzyme linked immunosorbent assays. We found that serum miR-21, miR-378 and miR-940 levels were significantly up-regulated immediately following an acute exercise while the rest were not changed. In addition, no robust correlation was identified between changes of these miRNAs and exercise capacity, muscle damage or inflammation. In conclusion, serum miR-21, miR-378, and miR-940 increase in response to an acute exhaustive exercise in CHF patients. Further studies are needed to clarify the potential use of circulating miRNAs as biomarkers of exercise adaptation in CHF patients, and if they have any use as prognostic markers of cardiovascular outcomes.

## INTRODUCTION

Congestive heart failure (CHF), a growing epidemic, is a major cause of hospitalizations, morbidity, and mortality [1]. In Western societies, the prevalence of CHF in the general population is 1–2% and increases to 10% among those aged over 75 and 20% among those aged over 80 [2]. The improvement in mortality from acute coronary syndromes due to more rapid revascularization has coincided with an increase in the late sequelae of myocardial infarction, namely CHF [1]. In addition, an increase in the age of the population has led to a marked increase in the incidence of CHF with preserved ejection fraction [1]. Together, the prevalence of CHF is increasing at a more rapid pace than any other cardiovascular diseases in the developed countries [1, 2]. This is likely to contribute to increased mortality, morbidity and health-care expenditures over the next few decades [1, 2].

Exercise training is an effective adjunct nonpharmacological treatment option for CHF patients not only to improve exercise capacity and quality of life, but also to decrease major cardiovascular events, including mortality and morbidity [3–5]. Therefore, exercise training has been recommended as an important cornerstone of therapy for CHF patients by ACC, AHA, and the Heart Failure Society of America [1, 6]. However, the underling cellular and molecular mechanism for benefical effects of exercise is still unclear [7].

MicroRNAs (miRNAs, miRs) are 19–22 nucleotides non-coding RNAs that negatively regulate gene expression at the post-transcriptional level via mRNA degradation or translational inhibition [8–10]. As a single miRNA can regulate hundreds of genes directly or indirectly, miRNAs have been central players of gene regulation [11]. Therefore, miRNAs participate in a variety of physiological and pathological processes. Dysregulation of miRNAs contributes to many diseases including cardiovascular diseases and cancers [12–15]. Recently, miRNAs have been found to be present in a stable form in the circulation and have been found to be dynamically regulated in response to physiological and pathological processes [16–19]. Intriguingly, circulating miRNAs may regulate gene expression in the target cells and tissues as a novel mode of cell-cell communication [20]. Dynamic regulation of circulating miRNAs during exercise in healthy persons and athletes has recently been documented, however, the response of circulating miRNAs to exercise in CHF patients is undetermined [21–25]. Here we investigate how specific circulating miRNAs with well-established roles in major adaptive processes is linked to exercise training in CHF patients. Specifically, we determined the expression levels’ changes of circulating miRNAs before and after an acute exhaustive exercise in CHF patients. We found that serum miR-21, miR-378, and miR-940 increased in response to an acute exhaustive exercise in CHF patients. However, no robust correlation was identified between changes of these miRNAs and exercise capacity, muscle damage or inflammation, indicating further studies using high-throughput circulating miRNAs screening techniques are highly needed to identify the potential use of circulating miRNAs as biomarkers of aerobic exercise capacity.

## RESULTS

### Subject characteristics

The cohort used in this study is the same as previously reported [26]. A total of 28 male HF participants were enrolled with a mean age of 59.07 ± 1.79 ys, height of 171.54 ± 1.05 cm, and body mass of 75.80 ± 1.43. The clinical characteristics for these subjects were shown in Table 1. The average EF was 47.68 ± 2.58%. The detailed echocardiographic parameters for these participants were indicated in Table 2.

**Table 1:** Clinical characteristics of participants

Clinical Parameters	Mean ± SEM
Age (years)	59.07 ± 1.79
Height (cm)	171.54 ± 1.05
Body mass (kg)	75.80 ± 1.43
BMI (kg/m^2^)	25.89 ± 0.51
Heart rate (beats/min)	71.89 ± 2.54
Work load (watt)	40.48 ± 2.95
VO_2_max (ml/min/kg)	16.84 ± 0.77
Systolic blood pressure (mmHg)	114.29 ± 3.45
Diastolic blood pressure (mmHg)	73.57 ± 2.44

**Table 2:** General echocardiographic indexes

Clinical Parameters	Mean ± SEM	Normal Values
Left ventricular end diastolic diameter (mm)	56.79 ± 1.77	35–56
Left ventricular end systolic diameter (mm)	42.07 ± 1.99	23–35
Interventricular septal thickness IVS (mm)	9.86 ± 0.26	6–11
Left ventricular posterior wall thickness (mm)	8.46 ± 0.29	6–11
Left atrial Dimension (mm)	46.89 ± 1.07	19–40
Pulmonary arterial systolic pressure (mmHg)	35.62 ± 2.01	18–30
Pulmonary artery diameter (mm)	26.25 ± 0.52	19–27
Ejection fraction (EF%)	47.68 ± 2.58	60–75
Aortic root dimension (mm)	34.46 ± 0.55	20–37

### Circulating miR-21, miR-378, and miR-940 increase in response to an acute exhaustive exercise in CHF patients

**Figure 1:**
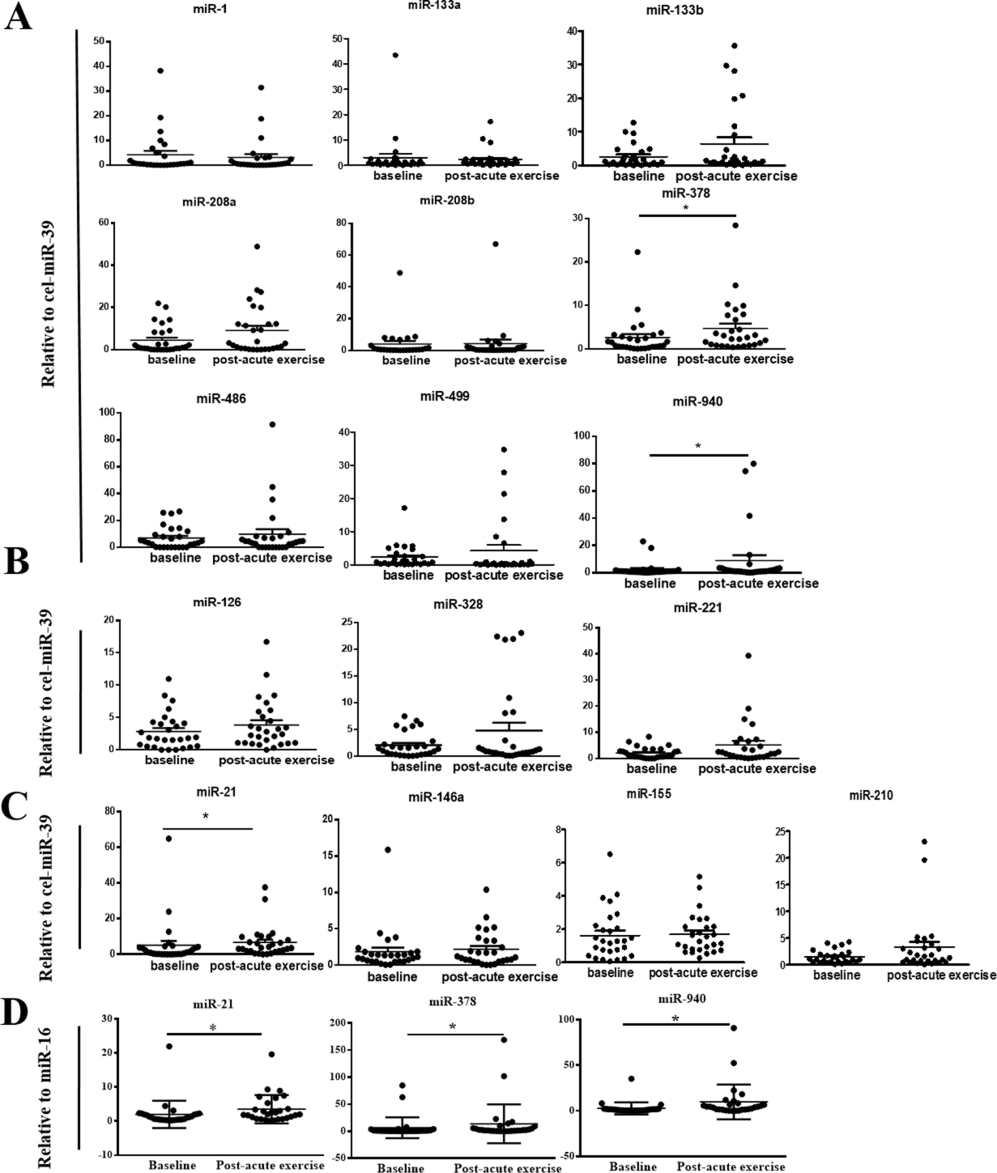
Changes in circulating microRNAs in response to an acute exhaustive exercise in chronic heart failure patients (**A**) Changes of cardiac or muscle specific/enriched miRNAs (miR-1, miR-133a, miR-133b, miR-499, miR-208a, miR-208b, miR-378, miR-486, and miR-940) when normalized to cel-miR-39; *compared to the baseline, *P* < 0.05. (**B**) Changes of miRNAs involved in angiogenesis (miR-328, miR-126, miR-221) when normalized to cel-miR-39; (**C**) Changes of miRNAs involved in ischemia adaptation and inflammation (miR-210, miR-21, miR-146a, miR-155) when normalized to cel-miR-39; *compared to the baseline, *P* < 0.05; (**D**) Changes of miR-21, miR-378 and miR-940 when normalized to miR-16; *compared to the baseline, *P* < 0.05.

The cardiac or muscle specific/enriched miRNAs including miR-1, miR-133a, miR-133b, miR-499, miR-208a, miR-208b, miR-378, miR-486, and miR-940 were determined. In addition, other miRNAs involved in angiogenesis (miR-328, miR-126, miR-221), inflammation (miR-21, miR-146a, miR-155), and ischemia adaptation (miR-210, miR-21, miR-146a) were also investigated. The majority of cardiac or muscle specific/enriched miRNAs except for miR-378, miR-486, and miR-940 displayed extremely low expression level as anticipated based on previous studies [23, 27]. Levels of serum miR-21, miR-378 and miR-940 were significantly up-regulated immediately following an acute exercise (Figure 1A–1C). In contrast, miR-1, miR-133a, miR-133b, miR-499, miR-208a, miR-208b, miR-486, miR-328, miR-126, miR-221, miR-146a, miR-155 and miR-210 were not changed following an exhaustive exercise (Figure 1A–1C). As at present, huge difference exist in choosing endogenous controls, it is recommend to have multiple endogenous controls to assure a robust finding regardless of the way of standardization [27]. Interestingly, when the values were normalized to miR-16, a similar tendency of serum levels of miR-21, miR-378 and miR-940 after an acute exercise was also found (Figure 1D). Collectively, these data consistently indicate that circulating miR-21, miR-378, and miR-940 increase in response to an acute exhaustive exercise in CHF patients.

### Correlations between changes of miR-21, miR-378, and miR-940 and exercise capacity, muscle damage or inflammation

**Figure 2:**
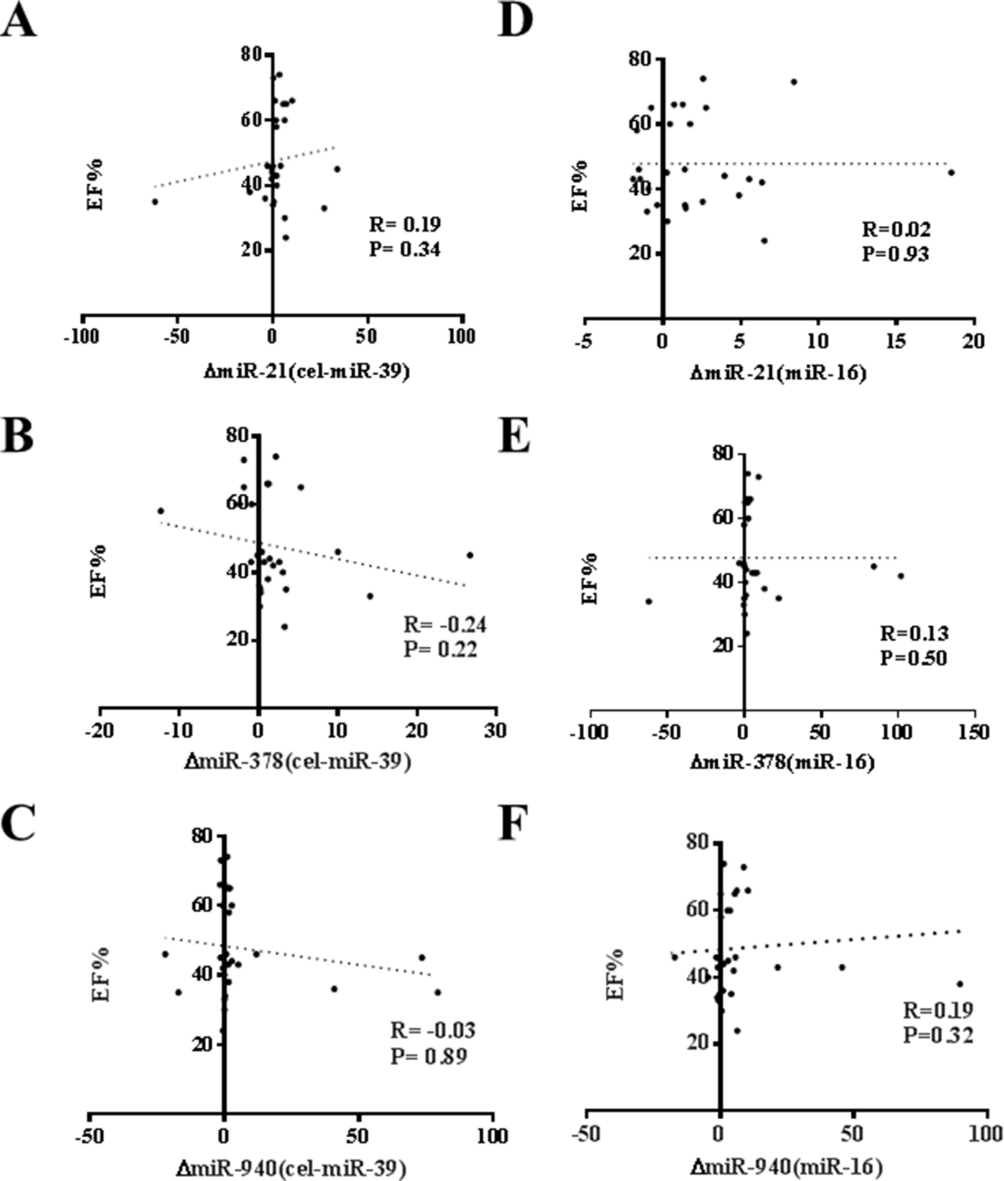
No correlation of changes of miR-21, miR-378 and miR-940 in exercise is observed with ejection fraction (EF%) (**A**) miR-21 (normalized to cel-miR-39); (**B**) miR-378 (normalized to cel-miR-39); (**C**) miR-940 (normalized to cel-miR-39); (**D**) miR-21 (normalized to miR-16); (**E**) miR-378 (normalized to miR-16); (**F**) miR-940 (normalized to miR-16).

**Figure 3:**
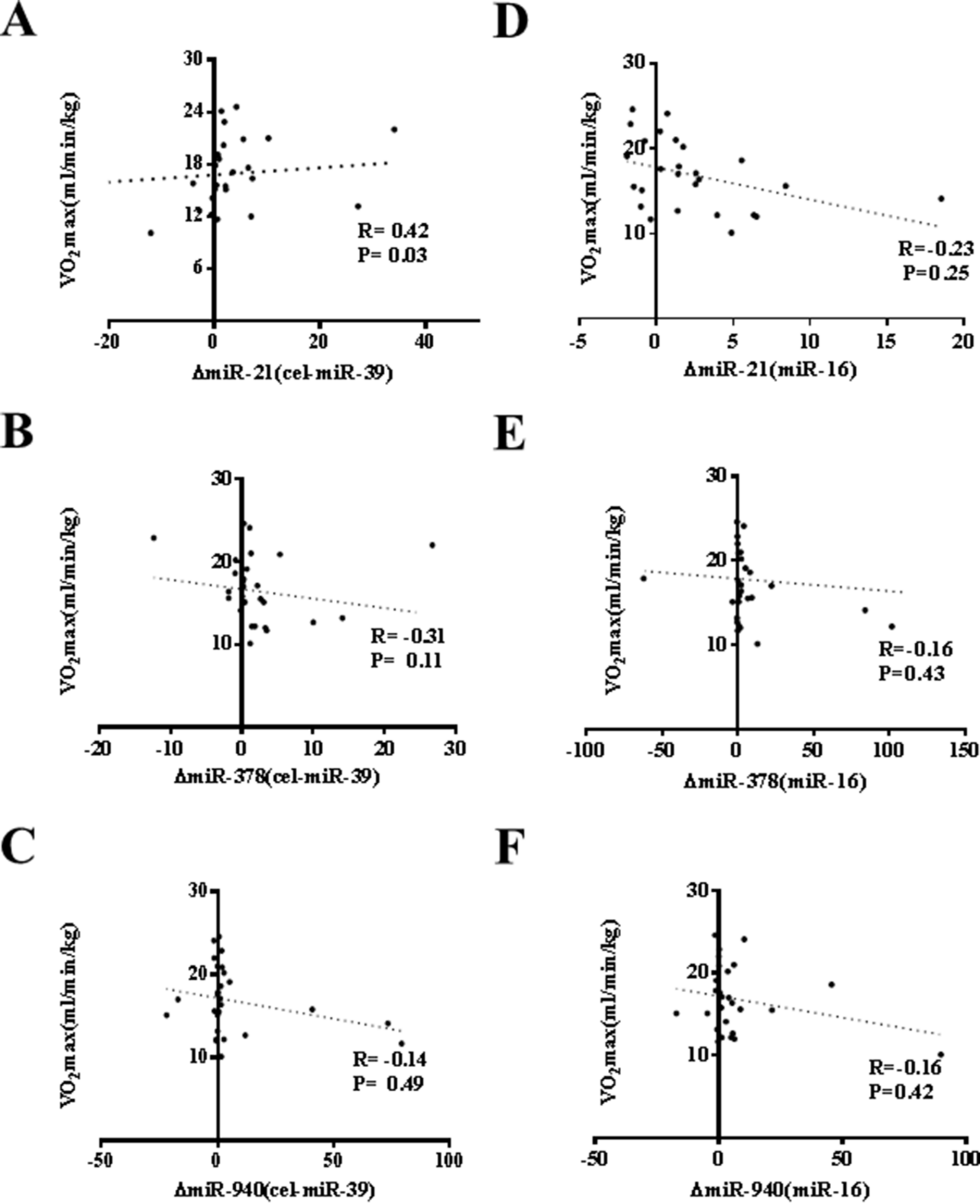
No robust correlation of changes of miR-21, miR-378 and miR-940 in exercise is observed with peak oxygen consumption (VO_2max_) (**A**) miR-21 (normalized to cel-miR-39). Note: A moderate correlation of changes of miR-21 in exercise is observed with VO_2max_; (**B**) miR-378 (normalized to cel-miR-39); (**C**) miR-940 (normalized to cel-miR-39); (**D**) miR-21 (normalized to miR-16); (**E**) miR-378 (normalized to miR-16); (**F**) miR-940 (normalized to miR-16).

**Figure 4:**
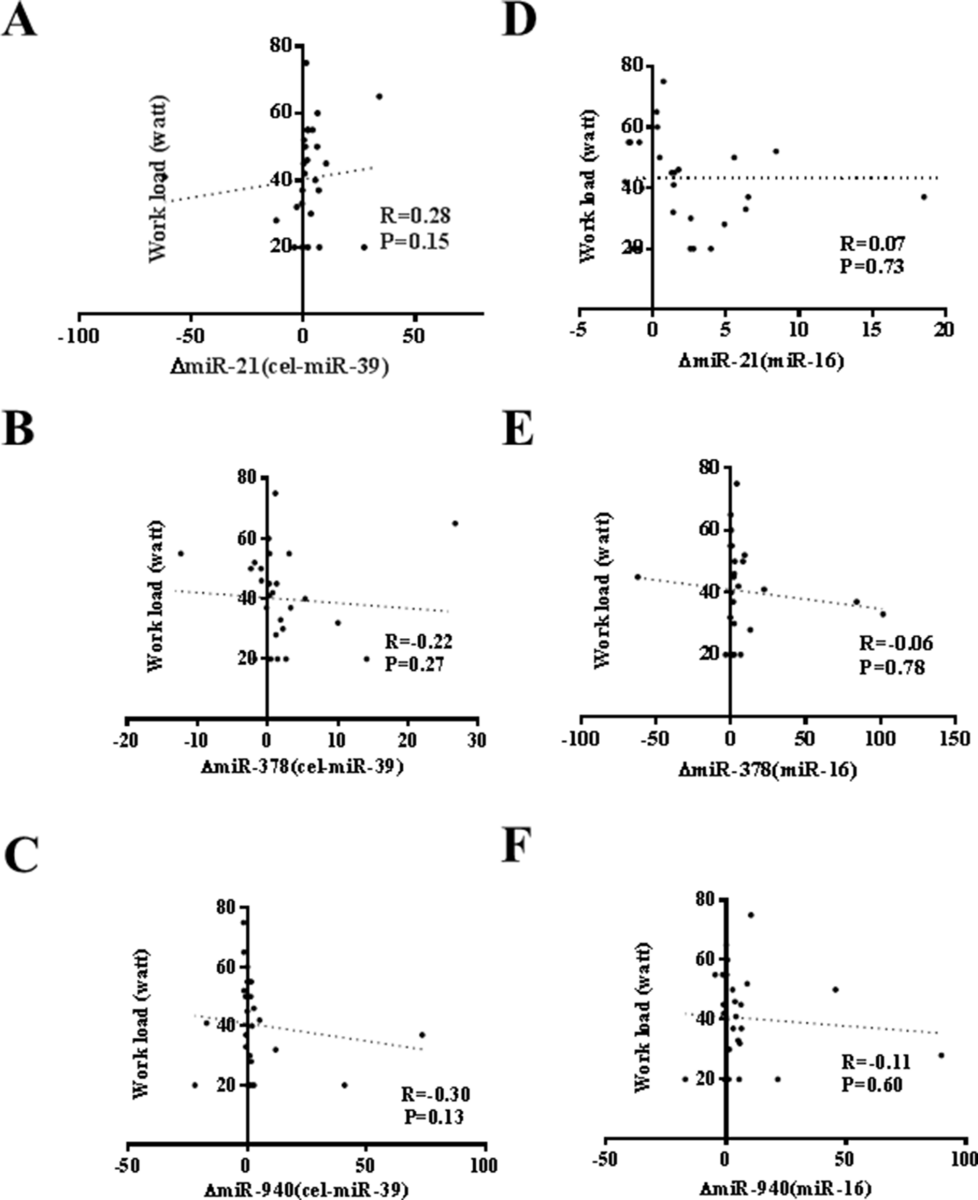
No correlation of changes of miR-21, miR-378 and miR-940 in exercise is observed with peak work load (**A**) miR-21 (normalized to cel-miR-39); (**B**) miR-378 (normalized to cel-miR-39); (**C**) miR-940 (normalized to cel-miR-39); (**D**) miR-21 (normalized to miR-16); (**E**) miR-378 (normalized to miR-16); (**F**) miR-940 (normalized to miR-16).

**Figure 5:**
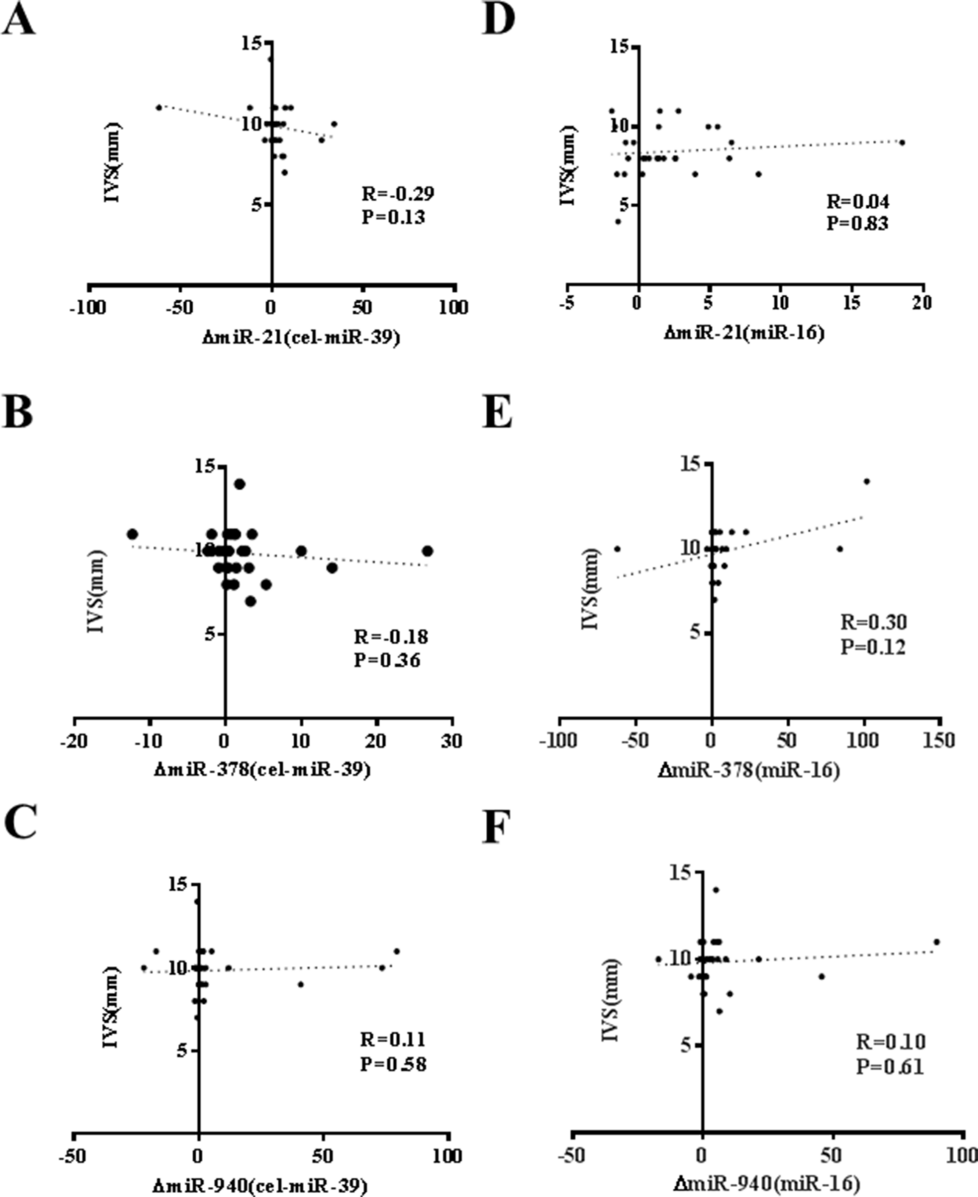
No correlation of changes of miR-21, miR-378 and miR-940 in exercise is observed with running speed at individual anaerobic lactate threshold (V_IAS_) (**A**) miR-21 (normalized to cel-miR-39); (**B**) miR-378 (normalized to cel-miR-39); (**C**) miR-940 (normalized to cel-miR-39); (**D**) miR-21 (normalized to miR-16); (**E**) miR-378 (normalized to miR-16); (**F**) miR-940 (normalized to miR-16).

An acute exercise in CHF patients increased the levels of CK, LDH, and NT-ProBNP while did not affect CK-MB, Tn-T and hs-CRP (Table 3). As that has been analyzed in athlete before and after a marathon run in a previous report [26], here we also correlated the increase of circulating miRNAs induced by exercise to makers of cardiac function and exercise capacity as well as indicators of muscle damage and inflammation. As a robust correlation would be consistently found no matter choose cel-miR-39 or miR-16 as endogenous controls, here we failed to report a robust correlation between changes of miR-21, miR-378, and miR-940 and cardiac function (Figure 2), exercise capacity (Figures 3–5), muscle damage or inflammation (Table 4), indicating further studies using miRNA arrays or RNA-seq are highly needed.

**Table 3:** Biochemical measurements

	Baseline	After acute exercise
**Destruction parameters**		
CK (ng/ml)	6.48 ± 1.12	11.20 ± 1.03*
LDH (U/l)	2355.98 ± 89.65	2598.94 ± 63.81*
**Cardiac markers**		
CKMB (ng/ml)	92.56 ± 13.05	80.60 ± 11.32
Troponin T (pg/ml)	42.74 ± 8.16	44.23 ± 8.60
NT-ProBNP (pg/ml)	193.38 ± 32.94	273.57 ± 45.68*
**Inflammatory marker**		
Hs-CRP (ng/ml)	9.845 ± 1.22	8.29 ± 0.90

Values are means ± SEM. CK, creatine kinase; LDH, lactate dehydrogenase; CK-MB, myocardial isoenzyme; Pro-BNP, pro-brain natriuretic peptide; hsCRP, high-sensitive C-reactive protein; *Significant difference between baseline/post-acute exercise.

**Table 4:** Correlations between changes of microRNAs and biochemical indexes

Δ miRNA		CK		LDH		CKMB		Troponin T		NT-ProBNP		hs-CRP	
		R	P	R	P	R	P	R	P	R	P	R	P
miR-21	cel-miR39	0.15	NS	0.39	0.04	0.06	NS	−0.12	NS	−0.33	NS	0.03	NS
	miR-16	−0.40	0.04	0.20	NS	0.01	NS	0.06	NS	0.39	0.04	0.03	NS
miR-378	cel-miR39	0.25	NS	−0.46	0.02	0.32	NS	−0.14	NS	−0.29	NS	0.35	NS
	miR-16	−0.3	NS	0.21	NS	−0.11	NS	−0.01	NS	0.33	NS	−0.12	NS
miR-940	cel-miR39	0.41	0.03	−0.21	NS	0.15	NS	0.06	NS	−0.28	NS	−0.06	NS
	miR-16	−0.20	NS	0.34	NS	0.01	NS	0.22	NS	0.22	NS	−0.22	NS

miR, microRNA; NS, not significant.

## DISCUSSION

This study was designed to investigate how specific circulating miRNAs are modulated by an acute exhaustive exercise in CHF patients, and if there are differences compared to exercise adaptation in healthy control subjects or athletes that have been previously published [21–28]. Here we reported that serum miR-21, miR-378, and miR-940 increased in response to an acute exhaustive exercise in CHF patients. We also showed an acute exercise in these CHF patients increased the levels of CK, LDH, and NT-ProBNP. Interestingly, no robust correlation is identified between the increase of circulating miRNAs induced by exercise to makers of cardiac function and exercise capacity as well as indicators of muscle damage and inflammation, indicating further studies using high-throughput circulating miRNAs screening techniques are highly needed. Moreover, the potential biological function of these dysregulated miRNAs responsible for beneficial effects of exercise training in CHF patients warrants further studied.

Several circulating miRNAs have been identified to respond to acute exhaustive exercise in healthy persons or athletes [21]. Specificially, circulating miR-1, miR-133, miR-499, miR-208, miR-146a, miR-221, miR-222, miR-338-3p, miR-330-3p, miR-223, miR-139-5p, miR-143, miR-21 and miR-149* were increased while miR-486, miR-106a, miR-30b, miR-151-5p, let-7i, miR-652 and miR-151-3p were robustly decreased after an acute exercise [21, 25, 28]. However, all these studies were conducted in healthy individuals or athletes. To the best of our knowledge, the present study is the first to identify serum miRNAs that change in response to exercise in CHF patients. Here we reported that serum miR-21, miR-378, and miR-940 increased in response to an acute exhaustive exercise in CHF patients while other miRNAs determined in this study were not changed, indicating that participants, exercise type, duration, and intensity might affect levels of circulating miRNAs [22]. In addition, these dysregulated miRNAs reported here are different from those reported in healthy control subjects or athletes, indicating a distinct exercise adaptation in CHF patients. Moreover, as most cardiac or muscle specific/enriched miRNAs were not changed in the present study, there must be distinct mechanisms of circulating miRNAs response to exercise rather than a general response to tissue damage.

As an initial “proof-of-concept” study, instead of using a more comprehensive but perhaps less reliable, high-throughput miRNA arrary technique, we used quantitative circulating miRNAs analysis checking selected miRNAs to decrease ‘false positive hits’. The selected miRNAs have potential biological relevance in exercise including angiogenesis, inflammation, and ischemia adaptation. Among the miRNAs analyzed here are cardiac or muscle specific/enriched miRNAs including miR-1, miR-133a, miR-133b, miR-499, miR-208a, miR-208b, miR-378, miR-486, and miR-940. Among these, miR-940 is a miRNA recently found to be enriched in human cardiac tissue and potentially contribute to human Tetralogy of Fallot [29]. However, little information is known about this miRNA. Here we provide novel insights that this miRNA might participate in the response to exercise.

Several limitations of the present study should be highlighted. Firstly, a major weakness of the present study is that only one time point was selected for miRNAs determination after exercise. Considering circulating miRNA changes are likely to involve different mechanisms including cellular release or changed transcription, it would be interesting to compared their expression at the very acute phase (e.g. minutes after exercise) and at the prolonged phase (hours to days after exercise). Secondly, though beyond the scope the this study, the source and potential biological function of miR-21, miR-378, and miR-940 warrant to be clarified in the future. These miRNAs might be carried in the serum by Ago proteins or membrane vesicles like exosome [20, 30–32]. As cardiac or muscle specific miRNAs including miR-1, miR-133a, miR-133b, miR-499, miR-208a and miR-208b are not increased in this study, we speculated that the increase of miR-21, miR-378, and miR-940 in serum is an active release process, which might affect the cells nearby or in distant tissues and integrate the physiological adaptation to exercise as a whole organism. Thirdly, it would be interesting to see what genes are also effected by this change in miRNAs. Fourthly, to acquire a more complete picture of exercise-induced circulating miRNAs, a more complete characterization including clearance of miRNAs reported in this study and others in a larger number of human subjects is highly needed. Finally, the current study examines circulating miRNAs in response to an acute exhaustive exercise in CHF patients. Informed conclusions regarding the dynamic regulation of circulating miRNAs after sustained aerobic exercise training can not be extrapolated at this time and need to be further studied. Finally, whether there is a prognostic role for these miRNAs in long-term outcomes is also undetermined.

In conclusion, serum miR-21, miR-378, and miR-940 increase in response to acute exhaustive exercise in CHF patients. Future studies aim at defining the potential use of circulating miRNAs as biomarkers of exercise training and a better understanding of the direct biological function of circulating miRNAs in adaptation to exercise training are highly needed.

## MATERIALS AND METHODS

### Participants

Approval was obtained from the ethics committee of Shanghai Tongji Hospital. All human investigation complied with the principles outlined in the Declaration of Helsinki. All participants provided written informed consent before participating in the study. Between September 2013 and March 2014, twenty-eight patients with CHF underwent a symptom-limited incremental cardiopulmonary exercise test on a bicycle ergometer (GE, USA) using a standardized exercise protocol of revised Ramp10 programs at Shanghai Tongji Hospital as previously reported [26]. Briefly, the patient was recorded at rest on the treadmill for 3 min. After that, the patient would cycle at 60 rpm for another 3 min without any resistance. Then the work was increased at a pre-set rate (starting from 20 J/s for 2 min and increased by 5J/s every 30s). The patient continued pedalling at 60 rpm throughout the test, until they were exhausted. All participants did not have the experience of conducting cardiopulmonary exercise test at least for six months and were abstained from any physiological exercise for 1 day before and were conduct an overnight fasting. On the morning of testing, participants were permitted and encouraged to drink water (400–800 ml) but without caloric or electrolyte content.

### Serum sampling and RNA isolation

Before and immediately after the cardiopulmonary exercise test, venous blood was collected in Serum Tubes (increased silica act clot activator, silicone-coated interior) and processed within 1 hour of collection. After a two-step centrifugation (820 × g for 10 min at 4°C, and then 16000 × g for 10 min at 4°C), the supernatant (serum) was transferred to RNase/DNase-free tubes and stored at −80°C until further analysis.

The total RNA was isolated from the serum using a mirVana PARIS isolation kit (Ambion, Austin, Texas) according to the manufacturer's instructions for serum samples without enrichment for small RNAs. Briefly, 400 μL of serum was used to extract the total RNA. Caenorhabditis elegans miR-39 (cel-miR-39) of 50 pmol/L was added as the spike-in control after the equal volume of denaturing solution was added. Each sample was eluted in 100 μL of RNAse-free water.

### Determination of circulating miRNAs levels

For quantitative miRNA analysis, Bulge-LoopTM miRNA qPCR Primer Sets (RiboBio) were used to detect selected miRNAs expressions by quantitative reverse transcription polymerase chain reactions (qRT-PCRs) with Takara SYBR Premix Ex Taq™ (TliRNaseH Plus) in the 7900HT Fast Real-Time PCR System as previously reported [26]. All qRT-PCR reactions were performed in triplicate, and the signal was collected at the end of every cycle. As there is no consensus on endogenous stable miRNAs in the circulation to act as house-keepers, the expression level of miRNAs in serum were normalized using spike-in cel-miR-39, which lacks sequence homology to human miRNAs. Besides that, miR-16, a representative miRNA enriched in serum, was also used in this study as an endogenous control.

### Biochemical measurements

Creatine Kinase (CK), Creatine Kinase MB Isoenzyme (CK-MB), Troponin T (Tn-T), N-Terminal Pro-Brain Natriuretic Peptide (NT-ProBNP) and high sensitive C Reactive Protein (hs-CRP) were determined by enzyme linked immunosorbent assays (ELISA) from Wuhan Xinqidi Biological Technology. Lactate dehydrogenase (LDH) was determined by ELISA kits from KeyGEN BioTECH.

### Statistical analysis

Participants characteristics data were shown as mean ± SD. For the analysis of qRT-PCR data, the relative expression level for each miRNA was calculated using the 2^−ΔΔCt^ method and the data were expressed as the mean ± SE. Paired samples were compared by Student's *t* test or Wilcoxon's matched pairs test as appropriate for data distribution. Correlation analyses between changes of circulating miRNAs before and after an acute exercise and exercise capacity, muscle damage or inflammation were performed using the Spearman's or Pearson's method as appropriate for data distribution. *P*-values less than 0.05 were considered to be statistically significant.
